# Benefits and Drawbacks of Harboring Plasmid pP32BP2, Identified in Arctic Psychrophilic Bacterium *Psychrobacter* sp. DAB_AL32B

**DOI:** 10.3390/ijms20082015

**Published:** 2019-04-24

**Authors:** Anna Ciok, Adrian Cegielski, Dariusz Bartosik, Lukasz Dziewit

**Affiliations:** Department of Bacterial Genetics, Institute of Microbiology, Faculty of Biology, University of Warsaw, Miecznikowa 1, 02-096 Warsaw, Poland; aciok@biol.uw.edu.pl (A.C.); cegielskiadrian@yahoo.com.au (A.C.); bartosik@biol.uw.edu.pl (D.B.)

**Keywords:** *Psychrobacter*, psychrophile, plasmid, Arctic, adhesion, biofilm formation, carnitine metabolism, trimethylamine

## Abstract

*Psychrobacter* sp. DAB_AL32B, originating from Spitsbergen island (Arctic), carries the large plasmid pP32BP2 (54,438 bp). Analysis of the pP32BP2 nucleotide sequence revealed the presence of three predicted phenotypic modules that comprise nearly 30% of the plasmid genome. These modules appear to be involved in fimbriae synthesis via the chaperone-usher pathway (FIM module) and the aerobic and anaerobic metabolism of carnitine (CAR and CAI modules, respectively). The FIM module was found to be functional in diverse hosts since it facilitated the attachment of bacterial cells to abiotic surfaces, enhancing biofilm formation. The CAI module did not show measurable activity in any of the tested strains. Interestingly, the CAR module enabled the enzymatic breakdown of carnitine, but this led to the formation of the toxic by-product trimethylamine, which inhibited bacterial growth. Thus, on the one hand, pP32BP2 can enhance biofilm formation, a highly advantageous feature in cold environments, while on the other, it may prevent bacterial growth under certain environmental conditions. The detrimental effect of harboring pP32BP2 (and its CAR module) seems to be conditional, since this replicon may also confer the ability to use carnitine as an alternative carbon source, although a pathway to utilize trimethylamine is most probably necessary to make this beneficial. Therefore, the phenotype determined by this CAR-containing plasmid depends on the metabolic background of the host strain.

## 1. Introduction

Bacteria of the genus *Psychrobacter* (class *Gammaproteobacteria*) are Gram-negative, non-motile, psychrotolerant, and halotolerant coccobacilli [1,2,3]. Currently (January 22, 2019), the genus includes 42 validated species, isolated mainly from permanently cold environments, including the Arctic and Antarctic regions [1,4,5,6,7]—frequently from the feces of various animals, e.g., sea birds and seals [4,8,9]. *Psychrobacter* spp. have also been detected in human tissues and blood, which suggests that some strains may be opportunistic pathogens [10,11].

Like most psychrophilic bacteria, *Psychrobacter* spp. preferentially grow at low temperatures (not exceeding 20 °C). The majority of strains are recognized as halotolerant or halophilic, since they are able to grow in the presence of NaCl at concentrations of up to 25% (*w*/*v*). These bacteria also tolerate high concentrations of potassium and magnesium [2,12,13,14]. Interestingly, the increased occurrence of *Psychrobacter* spp. in some environments may be linked to the presence of various harmful xenobiotics (e.g., diverse hydrocarbons), which can be utilized as carbon sources [15,16]. Such metabolic properties with adaptive value are often determined by mobile genetic elements, including plasmids. However, available reports concerning the role of plasmids in the adaptation of psychrophilic and psychrotolerant bacteria to extreme polar conditions are limited and rather fragmentary (e.g., [17,18,19,20]).

Currently (January 22, 2019), the complete nucleotide sequences of 63 *Psychrobacter* spp. plasmids are available in the GenBank database (NCBI). They range in size from 1.8 to 59.9 kb, but the majority (70%) are small cryptic replicons that do not exceed 15 kb in size. Interestingly, many *Psychrobacter* strains have a multireplicon genome composition and can contain up to seven plasmids, e.g., *Psychrobacter* sp. P11G5 [21]. As *Psychrobacter* spp. serve as model cold-active and biotechnologically-valuable microorganisms, some of their plasmids have been used for the construction of cloning vectors to enable genetic engineering of psychrophilic bacteria [22,23,24].

Only a few plasmids of *Psychrobacter* spp. have been recognized as the carriers of genes that assist adaptation to specific environmental conditions, e.g., (i) pKLH80 of *P. maritimus* MR29-12 conferring resistance to streptomycin, β-lactams, and tetracycline [20] and (ii) pP62BP1 of *Psychrobacter* sp. DAB_AL62B enabling catabolic breakdown of organic sulfates [25]. The number of reports describing *Psychrobacter* spp. plasmids is limited, so there is a dearth of knowledge about the biology and ecological role of these extrachromosomal replicons.

Previously, we described the general genomic content and chromosomally-encoded adaptive features of the psychrophilic plasmid-containing Arctic bacterium *Psychrobacter* sp. DAB_AL32B [8,22]. This strain was isolated from the feces of little auks (dovekie; *Alle alle*) collected on Spitsbergen island (a breeding colony situated on a mountain slope in Ariekammen Isbjornhamna Bay—Hornsund Fjord; 77°00′37.0″N, 15°31′49.5″E) [8]. DAB_AL32B is a psychrophile that can grow at temperatures of between 4 and 25 °C. It is able to utilize anthracene and exhibits low level resistance to As(V), Cr(VI), Cu(II), and Zn(II) [8].

The DAB_AL32B strain carries two circular plasmids: a small cryptic replicon pP32BP1 (4,599 bp) and a larger plasmid pP32BP2 (54,438 bp) [8,22]. In this study, we performed a detailed structural and functional analysis of pP32BP2, which revealed the antagonistic influence of this plasmid on the metabolic and physiological properties of its host strain.

## 2. Results and Discussion

### 2.1. General Features of Plasmid pP32BP2

**Figure 1:**
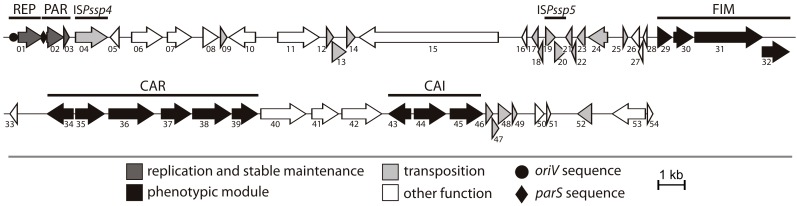
Linear map of plasmid pP32BP2. Arrows represent genes (numbered consecutively) and their transcriptional orientation. The predicted genetic modules are as follows: CAI—carnitine anaerobic metabolism, CAR—carnitine aerobic metabolism, FIM—fimbriae synthesis, PAR—partitioning, REP—replication. Insertion sequences IS*Pssp4* and IS*Pssp5* are indicated.

The available draft genomic data for *Psychrobacter* sp. DAB_AL32B [22] was insufficient to permit the assembly of the complete nucleotide sequence of plasmid pP32BP2. Therefore, plasmid DNA was isolated and sequenced *de novo* using Illumina technology. An analysis of the assembled sequence revealed that the size of pP32BP2 is 54,438 bp, with an average GC content of 39.9%, which is lower than that of the DAB_AL32B chromosome (41.9%) [22], but similar to the average value for other *Psychrobacter* plasmids (39.2%) (GenBank). The manual annotation of the pP32BP2 sequence identified 54 genes, which constitute 77.9% of the plasmid genome. Possible biological functions were assigned to 47 (87%) of them (Figure 1, Table S1).

A detailed analysis of the pP32BP2 sequence revealed that this plasmid contains several genetic modules (Figure 1). Besides the backbone modules responsible for replication (REP module) and stable maintenance (PAR module) functions, three additional conserved gene clusters were distinguished, comprising about 30% of the plasmid genome. These encode predicted proteins possibly involved in (i) fimbriae synthesis via the chaperone-usher pathway (FIM module), (ii) carnitine aerobic metabolism (CAR module), and (iii) carnitine anaerobic metabolism (CAI module). The functional characterization of these modules was performed and their genetic organization is reported in Figure 1.

Interestingly, pP32BP2 carries 18 genes that encode predicted transposases (Table S1). Seven are located within the DNA region between 20,039 and 22,050 bp, which is a putative hot spot for transposition. Comparative analyses (ISfinder database) revealed that only three transposase genes are complete, while the other 15 are pseudogenes, i.e., remnants of various transposase genes, that encode partial proteins. The accumulation of truncated transposons is not uncommon and has been observed in several other plasmid genomes [26,27].

Our analysis revealed that pP32BP2 carries two insertion sequences (ISs), as judged by the presence of (i) complete transposase genes, (ii) imperfect inverted repeat sequences (IR), representing the site of transposase binding and DNA cleavage, and (iii) direct repeat sequences (DR) adjacent to the IRs resulting from the duplication of the target site of transposition (Table 1). None of these elements carry additional passenger genes. The predicted ISs are novel elements, designated IS*Pssp4* and IS*Pssp5*, and their nucleotide sequences have been deposited in the ISfinder database. IS*Pssp4* (classified to the IS*256* family) carries a single transposase gene (*pP32BP2_p04*), while IS*Pssp5* (a member of the IS*427* group of the IS*5* family) contains two overlapping genes (*pP32BP2_p19* and *pP32BP2_p20*) that probably produce a functional transposase following a programmed translational frame-shift [28].

### 2.2. REP and PAR Modules—Plasmid Replication and Active Partitioning

The pP32BP2 backbone modules, REP (plasmid replication), and PAR (active partitioning), were described in detail previously [22]. In brief, the REP module structure is typical for iteron plasmids [29] and consists of a *repA* gene that encodes the replication initiation protein and an origin of replication (*oriV*) containing five direct repeats (DRs) and a pair of inverted repeats (IRs). The PAR module is comprised of three components: the *parA* and *parB* genes and a *parS* sequence preceding the putative *parAB* operon [22]. The REP and PAR modules were previously used to construct two *Psychrobacter*-specific cloning vectors—pPS-BR and pPS-NR [22]. In this study, the REP and PAR modules were cloned within the vector pABW1, which resulted in construct pABW1-REPPAR (Table 2). This recombinant plasmid was used to remove pP32BP2 from its host cells by means of the plasmid incompatibility phenomenon. As a result, the pP32BP2-less *Psychrobacter* sp. DAB_AL32B_PL was obtained and used for further analyses of the FIM, CAI, and CAR modules.

### 2.3. FIM Module—Cell Adhesion to Solid Surfaces and Biofilm Formation

**Figure 2:**
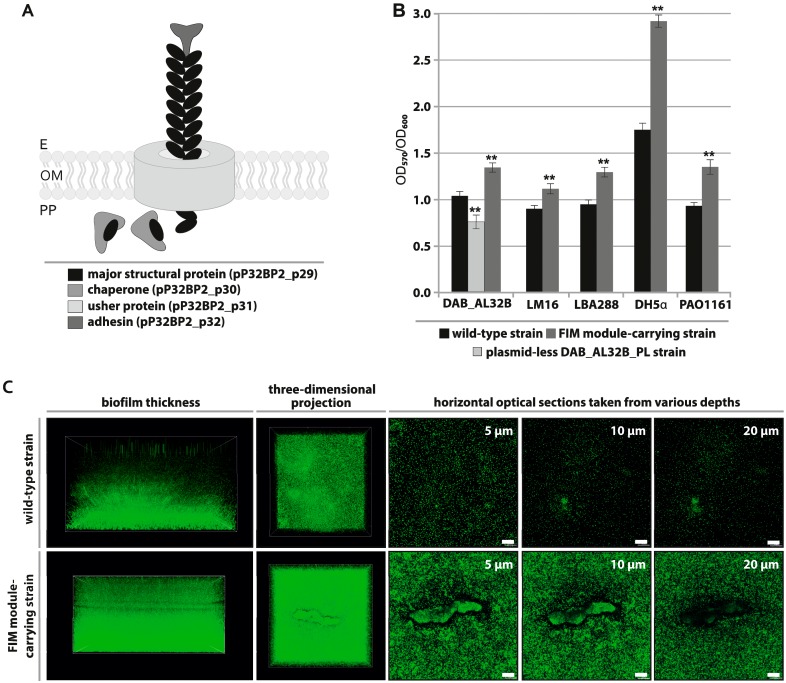
(**A**) Diagram of the predicted structure of fimbriae encoded by the FIM module of pP32BP2. E, OM, and PP—external, outer membrane, and periplasm, respectively. (**B**) Attachment of *Psychrobacter* spp. wild-type, pP32BP2-less, and plasmid-less carrying pBBR-Ps-FIM strains as well as heterologous wild-type and FIM module-carrying strains to the polystyrene surface of microtiter plates, measured by crystal violet staining. Error bars represent standard deviations. **—mean statistical significance *p* < 0.005, compared to adherence of the wild-type strain. LM16—*Achromobacter* sp. LM16, LBA288—*A. tumefaciens* LBA288, DH5α—*E. coli* DH5α, PAO1161—*P. aeruginosa* PAO1161. (**C**) Biofilm structure of wild-type and FIM module-carrying *P. aeruginosa* PAO1161 after 96 h of cultivation, visualized by scanning confocal laser microscopy. Horizontal optical sections were recorded at various depths (5, 10, and 20 μm) from the bottom of the dish. The scale bars represent 100 μm.

The FIM module of pP32BP2 is composed of four tandemly-arranged genes (*pP32BP2_p29*-*32*) (Figure 1). These encode structural proteins and components essential for fimbriae assembly via the chaperone-usher pathway (Figure 2A): (i) a major structural fimbrial subunit (COG3539; pP32BP2_p29), (ii) a chaperone (COG3121; pP32BP2_p30), (iii) an usher protein (COG3188; pP32BP2_p31), and (iv) an adhesin mediating attachment (pP32BP2_p32) (Table S1). Related gene clusters, displaying gene synteny and nucleotide sequence conservation were detected in the genomes of six *Psychrobacter* strains, i.e., PRwf-1, SHUES1, YP14, 1501(2011), UBA5136, and UBA3962 (GenBank account numbers CP000713, LXWA01000081, CP029789, AFHU01000208, DHYG01000067, and DGDC01000033, respectively). All but one of these FIM modules are located within the chromosome. The exception is that of the SHUES1 strain (draft genome), where the FIM-containing contig (Genbank account number LXWA01000081) is probably of plasmid origin, as it also carries a gene that encodes a putative plasmid replication initiation protein of the Rep_3 superfamily.

It is noteworthy that gene clusters homologous to the FIM module of pP32BP2 were also found in the genomes of many environmental and clinical isolates of the genus *Acinetobacter* (closely phylogenetically related to *Psychrobacter* spp.), e.g., *Acinetobacter junii* 65 that was isolated from limnetic water in the Novosibirsk region, Russia (GenBank account number CP019041) and *Acinetobacter haemolyticus* sz1652 obtained from the urinary tract of a hospital patient in Shenzhen, China (GenBank account number CP032135).

Fimbriae facilitate attachment to biotic and abiotic surfaces, colonization, and biofilm formation [30,31,32]. According to Hinsa-Leasure et al. [33], the majority of *Psychrobacter* strains are able to attach to solid surfaces, which is considered an advantageous feature in the cold and salty environments they inhabit. It was hypothesized that the attachment of bacterial cells in proximity to liquid water (a highly limited resource in freezing polar regions) increases bacterial survival in permafrost [33]. An enhanced attachment ability also facilitates colonization and biofilm formation. The ability to form biofilms is considered a highly advantageous feature, since the biofilm matrix protects cells against harmful environmental conditions (e.g., osmotic shock, desiccation, UV radiation), antimicrobial agents (e.g., antibiotics), and also enhances DNA acquisition [33,34,35,36].

To investigate whether the pP32BP2 FIM module is functional, we tested its contribution to the attachment of bacterial cells to an artificial solid surface (polystyrene microtiter plate). Attached cells of the strains DAB_AL32BR (wild-type), DAB_AL32B_PL (pP32BP2-less), and DAB_AL32B_PL carrying plasmid pBBR-Ps-FIM (vector pBBR1-MCS2 with cloned FIM module) were stained with crystal violet and analyzed as described in Materials and Methods.

The obtained results indicated that the FIM module does enhance the attachment of bacterial cells (Figure 2B). The attachment ability of the bacteria is significantly (*p* < 0.005) reduced in the absence of pP32BP2, and the reintroduction of the FIM module (within plasmid pBBR-Ps-FIM) restores the original phenotype. Compared to the wild-type strain DAB_AL32B, we observed stronger (*p* < 0.005) surface adhesion of the DAB_AL32B_PL strain carrying pBBR-Ps-FIM. This may be an effect of enhanced expression of the plasmid-borne FIM genes, as was previously seen in an analogous system by Schroll et al. [37].

Plasmids (and other mobile genetic elements) may spread via horizontal gene transfer (HGT) among bacteria belonging to diverse phylogenetic groups [38,39]. None of the genes identified within pP32BP2 are involved in conjugal transfer, nor are there sequences with similarity to known origins of conjugal transfer (*oriT*), which indicates that the plasmid is non-self-transmissible and non-mobilizable. Nonetheless, it is possible that, following host-cell death, this replicon or its fragments may be retrieved by various (non-*Psychrobacter*) microorganisms using other HGT mechanisms, e.g., transformation or transduction [40]. Therefore, the activity of the pP32BP2 FIM module was tested in four heterologous hosts belonging to different classes of *Proteobacteria* (*Alpha*, *Beta*, and *Gamma*). As shown in Figure 2B, all strains carrying plasmid pBBR-Ps-FIM displayed a statistically significant (*p* < 0.005) (Figure S1) enhanced attachment ability in comparison to the wild-type strains. This result suggests that this FIM cluster can be expressed in various *Proteobacteria* representing the most abundant inhabitants of polar environments [41,42,43].

The crystal violet staining assay is widely used to assess the ability of bacteria to form biofilms; however, it should only be considered a preliminary test of the adherence abilities of bacterial strains. Cell attachment is the first step in biofilm development. Since this initial analysis of bacterial adherence gave promising results, we then attempted to visualize the biofilm structures using scanning confocal laser microscopy (SCLM). Unlike the crystal violet assay, which examines attachment to a polystyrene surface, cells for imaging by SCLM were cultured on glass-bottomed dishes. Unfortunately, this difference in surface composition meant that biofilm structures could no longer be detected for four of the tested strains—*Psychrobacter* sp. DAB_AL32B, *Achromobacter* sp. LM16, *A. tumefaciens* LBA288, and *E. coli* DH5α. In these cases, we presumed that the weaker attachment to glass led to the loss of cells during the washing steps in sample preparation. However, in the case of *P. aeruginosa* PAO1161, we found that the FIM-carrying strain formed a significantly thicker biofilm than the wild-type (Figure 2C). This confirmed that the FIM module enhances biofilm formation and showed that cells of *P. aeruginosa* have the ability to attach to diverse abiotic surfaces. A more general conclusion from our results is that it is important to compare different basal surfaces in assays to analyze biofilm formation.

### 2.4. CAR Module—Aerobic Metabolism of Carnitine

**Figure 3:**
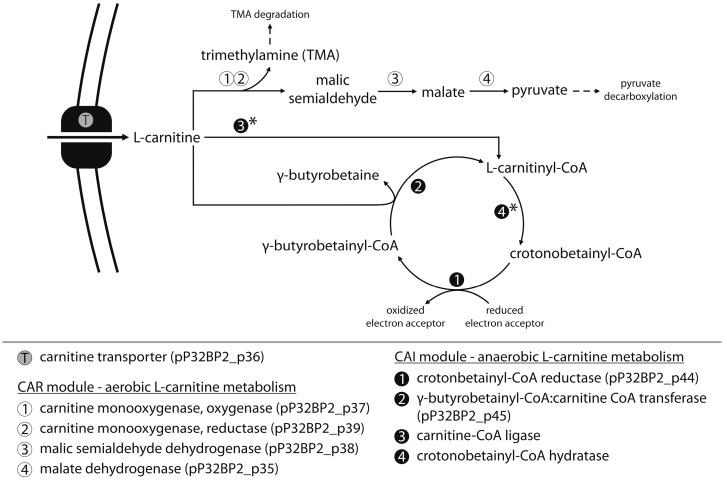
Predicted metabolic pathways determined by the CAR and CAI modules of pP32BP2. Asterisks mark enzymes whose genes are missing from the pP32BP2 genome. Solid arrow—reaction carried out by an enzyme encoded within the CAR module; dashed arrow—reaction carried out by enzymes not encoded within the CAR module.

The CAR module contains a cluster of five genes (*pP32BP2_p35-39*) preceded by a divergently oriented gene that encodes a transcriptional regulator of the LysR family (COG0583; *pP32BP2_p34*)—a putative regulator of CAR gene expression (Figure 1). Sequence similarity searches of the KEGG database allowed us to predict the biological function of the CAR-encoded proteins. Individual proteins may function as (i) a malate dehydrogenase (COG0473; *pP32BP2_p35*), (ii–iii) two-component carnitine monooxygenase (oxygenase [COG4638; *pP32BP2_p37*] and reductase [COG1018; *pP32BP2_p39*]), (iv) a malic semialdehyde dehydrogenase (COG1012; *pP32BP2_p38*), and (v) a BCCT-family transporter (glycine betaine/choline/carnitine transporter; TC: 2.A.15 [44]; *pP32BP2_p36*) (Table S1). Information obtained from the MetaCyc database [45] concerning these predicted proteins strongly suggested the involvement of the CAR module in L-carnitine transport and utilization under aerobic conditions (Figure 3).

**Figure 4:**
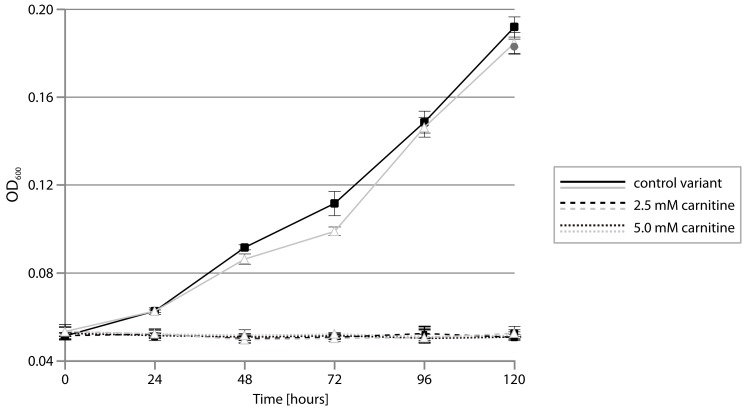
Growth of *Psychrobacter* sp. DAB_AL32B wild-type (■) and pP32BP2-less (Δ) strains on M9 minimal medium supplemented with sodium succinate (control variant; solid line) or carnitine (dashed and dotted lines) as the sole carbon source. Depending on the experimental variant, the M9 medium was supplemented with sodium succinate (0.5% *w*/*v*; control variant; solid lines) or carnitine (2.5 mM—dashed lines or 5 mM—dotted lines) as the sole carbon source. Triplicate cultures were grown in 50-mL tubes for 120 h at 20 °C with shaking under aerobic conditions. Every 24 h, the OD_600_ was measured. The mean values for three replicate cultures are plotted with error bars representing the standard deviations.

To test whether plasmid pP32BP2 enables the utilization of carnitine, the wild-type (DAB_AL32BR) strain and its pP32BP2-less derivative (DAB_AL32B_PL) were cultivated in a minimal medium supplemented with carnitine as the sole carbon source. We failed to observe changes in the optical density of the bacterial cultures, regardless of the concentration of carnitine in the medium, which demonstrated an absence of growth (Figure 4). However, a noticeable scent of trimethylamine (TMA; an intermediate in carnitine breakdown; Figure 3) was detected in the cultures of the wild-type strain. The concentration of trimethylamine, determined using the Folin-Ciocalteu phenol reagent assay, was 5.46 ± 0.35 mM (after 120 h of culture in a medium with an initial carnitine concentration of 5 mM). In contrast, no TMA was produced in the pP32BP2-less strain cultures. This indicated that carnitine breakdown occurs and trimethylamine is released only in the presence of pP32BP2.

To confirm that the trimethylamine originates from the utilization of carnitine, conferred by the presence of CAR, this module was cloned into vector pBBR-Ps (Table 2), and the resulting plasmid (pBBR-Ps-CAR) was introduced into the strain DAB_AL32B_PL (pP32BP2-less). As shown in Figure 1, three additional genes are located between the predicted CAR and CAI modules, encoding the following putative proteins: (i) large subunit of acetolactate synthase (COG0028; pP32BP2_p40), (ii) NADPH:quinone oxidoreductase (COG0604; pP32BP2_p41), and (iii) betaine aldehyde dehydrogenase (COG1012; pP32BP2_p42) (Figure 1, Table S1). Since it is possible that these enzymes might affect carnitine breakdown, their genes (*pP32BP2_p40-42*) were also included in the plasmid pBBR-Ps-CAR. When the strain DAB_AL32B_PL (pBBR-Ps-CAR) was cultivated in a minimal medium supplemented with carnitine as the sole carbon source, no growth was observed and trimethylamine was detected, as in the case of the wild-type strain. This confirmed that the TMA is produced by the activity of the CAR module.

To check whether the lack of growth on carnitine was a consequence of a production of toxic TMA, we first carefully inspected the genome of the DAB_AL32B strain for the presence of genes that encode enzymes responsible for trimethylamine utilization. This led to the identification of a putative *tmm*-like gene (GenBank account number OXL21465), sharing 58% aa identity with an experimentally-tested trimethylamine monooxygenase from *Paracoccus aminophilus* JCM 7686 [46]. Trimethylamine monooxygenases catalyze the transformation of trimethylamine into trimethylamine *N*-oxide (TMAO), which may function as an osmoprotectant in bacteria [47]. However, in the DAB_AL32B genome, we did not find other genes that were shown to be obligatory for further transformation of TMAO, including *tdm* gene encoding trimethylamine-*N*-oxide demethylase [48]. The *tdm* genes are absent in all other currently available *Psychrobacter* genomes (NCBI). Interestingly, genes that encode enzymes crucial for TMA utilization have been found in the genomes of *Acinetobacter* spp. that are closely phylogenetically related to *Psychrobacter* spp. Moreover, *Acinetobacter* spp. frequently harbor similar CAR modules and can exploit these gene clusters for the utilization of carnitine as a sole carbon source under aerobic conditions [49]. As the strain DAB_AL32B is apparently unable to use TMA as a carbon source due to lack of the appropriate genes, we tested whether malate, a second product of carnitine breakdown (Figure 3), is a suitable substrate for bacterial growth. DAB_AL32B was able to utilize this compound, so the lack of growth of this strain in a minimal medium containing carnitine is not a consequence of the absence of an accessible carbon source.

**Figure 5:**
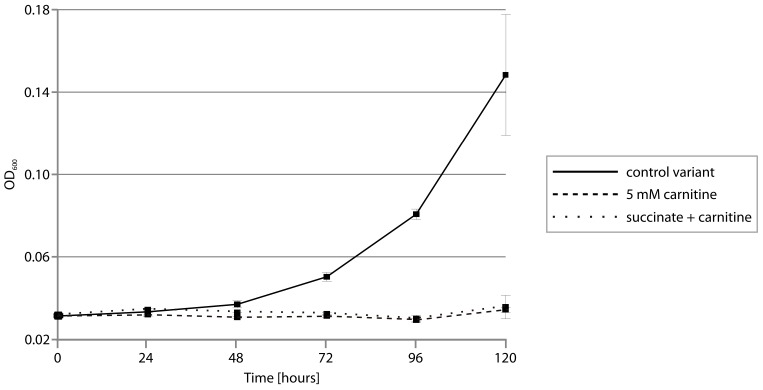
Growth of *Psychrobacter* sp. DAB_AL32B on sodium succinate (control variant, solid line), carnitine (5 mM) (dashed line), and sodium succinate plus carnitine (5 mM) (dotted line) as carbon sources. The mean values for three replicate cultures are plotted with error bars representing the standard deviations.

To verify the presumed toxic effect of carnitine utilization on the strain DAB_AL32B, we next checked its ability to grow in a minimal medium containing a mixture of two carbon sources, i.e., sodium succinate (previously shown to be optimal for this bacterium) together with carnitine. Again, we did not observe any change in the optical density of the cultures (Figure 5), indicating a lack of growth. In addition, the scent of trimethylamine was detected, and the presence of TMA was confirmed using the Folin-Ciocalteu phenol reagent assay. This result indicated that the breakdown of carnitine mediated by CAR releases a toxic compound, i.e., TMA, that prevents bacterial growth.

To our knowledge, there have been no reports concerning *Psychrobacter* growth on carnitine as a carbon source. When analyzing genomic sequences of *Psychrobacter* spp. (GenBank database), we did not identify any complete gene cluster that encodes enzymes responsible for aerobic carnitine utilization, i.e., complete CAR modules. Nonetheless, four *Psychrobacter* strains, i.e., *P. alimentarius* PAMC 27889, *P. fozii* CECT 5889, *Psychrobacter* sp. JCM 18900, and *Psychrobacter* sp. 4Bb (GenBank account numbers CP014945, QJSU01000003, BAWG01000006, and PJAS01000001, respectively) carry gene clusters that encode proteins (i.e., carnitine transporter, oxygenase component of carnitine monooxygenase, and malic semialdehyde dehydrogenase, respectively) homologous to pP32BP2_p36-38 (Figure S2). Unlike in DAB_AL32B, these loci have the gene that encodes the carnitine transporter divergently oriented in relation to the other two genes. A detailed analysis of the sequence contigs carrying these gene clusters suggest that they might be of chromosomal origin.

The ability to metabolize carnitine has been experimentally confirmed for three representatives of the genus *Acinetobacter*, i.e., *A. baumannii* ATCC19606, *A. calcoaceticus* ATCC39647, and *A. calcoaceticus* 69-V, but conserved gene clusters potentially involved in carnitine metabolism were also identified in the genomes of several other *Acinetobacter* spp., representing human gut microbiota [49,50,51]. In fact, gene clusters with strong similarity to the pP32BP2 CAR module are ubiquitous in both environmental and clinical *Acinetobacter* isolates, e.g., *A. oleivorans* KCJK7897 is isolated from soil in the USA (GenBank account number QAYN01000016), *A. harbinensis* HITLi from river water in China (GenBank account number JXBK01000002), and *A. baumannii* ATCC19606 from a human subject (GenBank account number GG704573) (Figure S2).

An aerobic carnitine degradation pathway (i.e., CAR module) seems not to occur in *Psychrobacter* spp. and appears to be a unique feature of plasmid pP32BP2. We speculate that this plasmid-borne CAR module may have been acquired via HGT from bacterium (possibly TMA-utilizing) representing other genus, e.g., closely related *Acinetobacter* (whose representatives were also isolated from the same soil sample like the DAB_AL32B strain [8]).

### 2.5. CAI Module—Anaerobic Metabolism of Carnitine

The CAI gene cluster, the second metabolic module located within plasmid pP32BP2, is composed of three genes (*pP32BP2_p43-45*) that encode the following putative proteins: (i) a transcriptional regulator of the IclR family (COG1414; pP32BP2_p43), (ii) crotonbetainyl-CoA reductase (COG1960; pP32BP2_p44), and (iii) γ-butyrobetainyl-CoA:carnitine CoA transferase (COG1804; pP32BP2_p45) (Figure 1, Table S1). The two enzymes exhibit homology to their respective counterparts (CaiA and CaiB) encoded within the *caiTABCDEF* operon of *E. coli* K-12 substr. MG1655 (GenBank account number U00096.3).

The CAI modules described to date encode proteins involved in carnitine metabolism under anaerobic conditions. Various members of the *Enterobacteriaceae* (e.g., *E. coli*, *Salmonella enterica* serovar Typhimurium, and *Proteus vulgaris*) are able to metabolize L-carnitine via crotonobetaine to γ-butyrobetaine (so-called L-carnitine degradation pathway I) [45]. Crotonobetaine serves as an external electron acceptor and enables bacterial growth under anaerobic conditions.

The pathway substrate, L-carnitine, enters the cell via the CaiT transporter (the BCCT transporter family) [50,52]. Since the CAI module of pP32BP2 does not encode any transporter, we presume that L-carnitine:γ-butyrobetaine antiport is mediated by BCCT family transporters encoded by the DAB_AL32B chromosome.

The L-carnitine degradation pathway I requires four enzymes (CaiABCD). Related enzymes are encoded in the DAB_AL32B chromosome, but they are not organized in one cluster, which may suggest that the observed sequence similarity is irrelevant. Plasmid pP32BP2 encodes only two proteins, CaiA and CaiB (homologous to CaiA and CaiB of *E. coli*), which means that this plasmid-borne CAI module is incomplete (Figure 3). Possibly, the CaiCD-encoding genes have been lost from the plasmid as a result of transposition events. Notably, transposase gene remnants are present downstream of *pP32BP2_p45* (Figure 1). However, it is still possible that the chromosomally-encoded CaiC- and CaiD-like proteins may complete the truncated plasmid-encoded degradation pathway.

To verify this hypothesis, wild-type and pP32BP2-less *Psychrobacter* sp. strains were cultivated under anaerobic conditions in the presence of sodium succinate (as carbon source) and L-carnitine. No growth was observed in any experimental variant (Figure S3), which confirmed that the CAI module is incomplete and that the identified chromosomal genes did not permit the use of carnitine as an external electron acceptor.

It is important to mention that growth of *Psychrobacter* spp. under anaerobic conditions has been experimentally demonstrated for only two strains, *P. aquimaris* SW-210^T^ and *P. namhaensis* SW-242^T^; thus, this ability may not be common to the entire genus [53].

## 3. Materials and Methods

### 3.1. Bacterial Strains, Plasmids and Culture Conditions

The following bacterial strains were used: *Achromobacter* sp. LM16R [54], *Agrobacterium tumefaciens* LBA288 [55], *Escherichia coli* DH5α [56], *Pseudomonas aeruginosa* PAO1161 [57], *Psychrobacter* sp. DAB_AL32B [8], *Psychrobacter* sp. DAB_AL32BR (Rif^r^-derivative of DAB_AL32B) [23], and—constructed in this study—*Psychrobacter* sp. DAB_AL32B_PL (i.e., derivative of DAB_AL32BR denuded of plasmid pP32BP2). Strains were grown at 20 °C (*Psychrobacter* spp.), 30 °C (*Achromobacter* sp. and *A. tumefaciens*), or 37 °C (*E. coli* and *P. aeruginosa*). Depending on the experiment, strains were cultivated on LB (lysogeny broth) or the following minimal media: (i) a M63 medium for *P. aeruginosa* (2 g/L (NH_4_)_2_SO_4_; 13.6 g/L KH_2_PO_4_; 0.5 mg/L FeSO_4_x7H_2_O; 0.246 g/L MgSO_4_x7H_2_O) supplemented with 0.2% (*w*/*v*) glucose, and (ii) a M9 medium for other strains (6 g/L Na_2_HPO_4_; 3 g/L KH_2_PO_4_; 1 g/L NH_4_Cl; 0.5 g/L NaCl; 0.12 g/L MgSO_4_; 0.01 g/L CaCl_2_) supplemented with 0.5% (*w*/*v*) sodium succinate (for *Psychrobacter* spp.) or 0.2% (*w/v*) glucose (for *Achromobacter* sp.*, A. tumefaciens*, and *E. coli).* The media were solidified by the addition of 1.5% (*w*/*v*) agar. Where necessary, media were supplemented with X-gal, IPTG and antibiotics: kanamycin (50 μg/mL for *A. tumefaciens*, *E. coli,* and *Psychrobacter* spp. or 500 μg/mL for *Achromobacter* sp. and *P. aeruginosa*) and rifampin (50 μg/mL). Plasmids used and constructed in this study are listed in Table 2.

### 3.2. DNA Manipulations and Introduction of Plasmid DNA into Bacterial Cells

Plasmid pP32BP2 DNA was isolated using a large-scale alkaline extraction method and purified by CsCl-ethidium bromide gradient centrifugation [62]. Other plasmid DNAs were isolated using a GeneMATRIX Plasmid Miniprep DNA Purification Kit (EURx, Gdansk, Poland) or a classical alkaline lysis procedure [63]. Routine DNA manipulations were carried out using standard methods [62]. DNA was amplified by PCR using a KAPA HiFi PCR Kit and appropriate primer pairs (Table S2). DNA amplification was performed using a Mastercycler (Eppendorf, Hamburg, Germany). Each thermocycle started with an initial denaturation at 95 °C for 3 min followed by 30 cycles of denaturation at 98 °C for 20 s, annealing at 62 to 65 °C (depending on the primer pair) for 15 s, extension at 72 °C for 1 min/kb, and finished with a final extension at 72 °C for 1 min/kb. PCR-amplified DNA fragments were then cloned in the pABW1 or pBBR1MCS-2 vectors.

To clone isolated CAR and FIM modules, two libraries of BamHI and EcoRI restriction fragments of pP32BP2 were prepared in vector pBGS18. Plasmid clones carrying fragments comprising the CAR or FIM modules were used in further steps to construct pBBR-Ps-CAR, pBBR-FIM and pBBR-Ps-FIM.

The obtained derivatives of pABW1 and pBBR1MCS-2 were introduced into *Achromobacter* sp. LM16R, *A. tumefaciens* LBA288, *Psychrobacter* sp. DAB_AL32BR, and *Psychrobacter* sp. DAB_AL32B_PL by triparental mating [64], into DAB_AL32BR and DAB_AL32B_PL by electroporation [65] and into *E. coli* and *P. aeruginosa* by chemical transformation [66,67].

### 3.3. Construction of Plasmid-Less Psychrobacter sp. DAB_AL32B

To remove the naturally occurring plasmid pP32BP2 from *Psychrobacter* sp. DAB_AL32B, a target-oriented replicon curing technique was applied [68]. A DNA fragment carrying the REP and PAR modules of pP32BP2 was amplified by PCR, cloned into vector pABW1 (resulting in plasmid pABW1-REPPAR), and introduced into strain DAB_AL32BR by electroporation. Several colonies were then screened for the presence of pP32BP2 by (i) DNA isolation and comparing plasmid profiles with the wild-type strain, and (ii) PCR to detect selected genes of pP32BP2 (Table S2). A clone denuded of plasmid pP32BP2 was selected, and a plasmid pABW1-REPPAR was removed from this strain in the following manner. A liquid culture was grown, and every 24 h this was diluted (1:1000) in a fresh medium lacking antibiotics. After 3 days, culture dilutions were plated onto a solid medium without antibiotic selection. About 50 colonies were tested for kanamycin resistance conferred by the presence of vector pABW1-REPPAR. Clones able to grow only on plates without antibiotic were checked for the presence of pABW1-REPPAR by applying the methods described above. The obtained plasmid-less strain, lacking both pP32BP2 and pABW1-REPPAR, was named DAB_AL32B_PL.

### 3.4. DNA Sequencing

The complete nucleotide sequence of plasmid pP32BP2 was determined in the DNA Sequencing and Oligonucleotide Synthesis Laboratory at the Institute of Biochemistry and Biophysics, Polish Academy of Sciences (Warsaw, Poland). The plasmid was sequenced using an Illumina MiSeq instrument in paired-end mode with a v3 chemistry kit (Illumina, San Diego, CA, USA). The obtained sequence reads were filtered for quality and assembled using Newbler v3.0 software (Roche, Basel, Switzerland). Final gap closure was performed by capillary sequencing of PCR amplicons using an ABI3730xl DNA Analyser (Applied Biosystems, Waltham, MA, USA).

All PCR products obtained in this study were cloned into vectors pABW1 or pBBR1MCS-2 and then sequenced using an ABI3730xl DNA Analyser (Applied Biosystems) to check for errors. Where necessary, primer walking was employed to obtain the complete nucleotide sequence of DNA fragments.

### 3.5. Bioinformatic Analyses

Plasmid DNA sequences were manually annotated using Artemis software [69]. Similarity searches were performed using the BLAST programs [70] with the NCBI Conserved Domains Database [71] (available online: http://blast.ncbi.nlm.nih.gov/Blast.cgi) and Pfam [72]. EC numbers were assigned using the KEGG database [73]. Metabolic pathways were screened using the MetaCyc database [45]. Insertion sequences were analyzed using the ISfinder database [74].

### 3.6. Testing Bacterial Adherence to Artificial Surfaces

To test bacterial adherence to artificial surfaces, a modified crystal violet staining method was used [75]. Bacteria were cultivated overnight in a minimal medium at the optimum temperature and then harvested by centrifugation, washed three times with saline (0.8% *w*/*v*), and used to inoculate a fresh medium to an OD_600_ of 0.05 ± 0.005. Samples of 200 μL of the cell suspensions (three biological replicates, each with six technical replicates) were transferred to the wells of sterile 96-well plates, and these were incubated at the required temperature without shaking. After 24 h (for *Achromobacter* sp., *A. tumefaciens*, *E. coli,* and *P. aeruginosa*) or 48 h (*Psychrobacter* spp.) the OD_600_ of the cultures was measured using a Sunrise^TM^ plate reader (with Magellan software; Tecan, Männedorf, Switzerland). The medium containing unbound cells was then removed, and all wells were rinsed twice with saline solution and dried at 37 °C for about 15 min. Attached bacteria were stained by adding 0.1% (*w*/*v*) crystal violet (200 μL/well) and incubating the plates at room temperature. After 10 min, the stain was removed, and the wells rinsed twice with saline solution and dried again at 37 °C. The dried stained biofilms were then dissolved by adding 95% ethanol (200 μL/well) and incubated for 10 min. The OD_570_ of the obtained suspension was measured using a Sunrise^TM^ plate reader. Biofilm formation was quantified by calculating the OD_570_/OD_600_ ratio. The statistical significance of obtained results was determined by the Mann-Whitney U test (Figure S1).

### 3.7. Live Cell Confocal Microscopy (Biofilm Analysis)

Scanning Confocal Laser Microscopy (SCLM) was applied to image live cells in biofilms [76]. Bacteria were cultivated on glass-bottomed dishes (35 mm diameter, 20 mm glass diameter, number 1.5 coverslip; MatTek Corpor, Ashland, OR, USA). Initial cultures were grown in an appropriate minimal medium under optimal conditions. The cells were then harvested by centrifugation, washed three times with saline (0.8% *w*/*v*), and used to inoculate a fresh medium to an OD_600_ of 0.06 ± 0.005. A volume of 4 mL of each cell suspension was transferred to a glass-bottomed dish and incubated without shaking at the optimum temperature for 72 h (for *Achromobacter* sp., *A. tumefaciens*, and *E. coli*) or 96 h (*P. aeruginosa* and *Psychrobacter* sp.). The medium was removed from the dish, and the biofilm that had developed on the bottom was washed three times with 10 mM MgSO_4_ and then stained with acridine orange solution (10 μg/mL in 10 mM MgSO_4_) (Sigma, Basel, Switzerland). After 30 min, the stain was removed, and the biofilm was rinsed twice with 10 mM MgSO_4_. Confocal microscopy was then performed using a Nikon Eclipse Ti (A1) microscope equipped with a ×60, 1.4 NA oil immersion phase-contrast lens (Nikon Corporation, Tokyo, Japan). An argon laser with a maximum-emission line at 488 nm was used as the excitation source. Horizontal optical thin sections were collected at 0.21-μm intervals from the outer surface of the biofilm to the bottom of the glass plate. These images were captured using NIS-ELEMENTS interactive software (Nikon Corporation), and three-dimensional reconstructions were created.

### 3.8. Testing Psychrobacter spp. Growth on Carnitine as the Sole Carbon Source Under Aerobic Conditions

Cultures of various *Psychrobacter* strains were grown in a M9 minimal medium supplemented with sodium succinate (0.5% *w*/*v*) as the carbon source. The cells were then harvested by centrifugation, washed three times with saline (0.8% *w*/*v*), and used to inoculate a fresh medium to an OD_600 of_ 0.05 ± 0.005. Depending on the experimental variant, the M9 medium was supplemented with sodium succinate (0.5% *w*/*v*; control) or carnitine (2.5 or 5 mM) as the sole carbon source. Triplicate 10 mL cultures were grown in 50-mL tubes for 120 h at 20 °C with shaking. Every 24 h, 200 μL of each culture was taken, the OD_600_ measured using a Sunrise^TM^ plate reader, and growth curves were plotted.

### 3.9. Determination of Trimethylamine Concentration

The trimethylamine concentration in bacterial cultures was determined using Folin-Ciocalteu phenol reagent according the procedure described by Ikawa et al. [77].

### 3.10. Testing Anaerobic Growth of Psychrobacter spp.

Cultures of *Psychrobacter* strains were grown in a M9 minimal medium supplemented with sodium succinate (0.5% *w*/*v*) as the carbon source. The cells were then harvested by centrifugation, washed three times with saline (0.8% *w*/*v*), and used to inoculate fresh M9 supplemented with sodium succinate (0.5% *w*/*v*) as carbon source and, depending on the experimental variant, 2.5 or 5.0 mM L-carnitine. Cultures of 80 mL were propagated anaerobically in 100-mL serum bottles (Sigma). Prior to inoculation, the desired medium was added to the bottle and this was then flushed with CO_2_:N_2_ (ratio 20:80). The bottles were then corked with silicon stoppers secured by aluminum crimp seals. Bacterial cells were introduced into the sealed bottles via the stopper using a syringe plus needle to obtain an initial OD_600_ of 0.06 ± 0.005. The cultures were grown for 7 days at 20 °C without shaking. To plot growth curves, 0.5 mL of each culture was collected on days 0, 1, 3, 5, and 7, and the OD_600_ measured using a Sunrise^TM^ plate reader. Each experimental variant was run in triplicate.

### 3.11. Nucleotide Sequence Accession Numbers

The nucleotide sequence of plasmid pP32BP2 was deposited in the GenBank (NCBI) database with the accession number MK422609. Sequences of IS*Pssp4* and IS*Pssp5* were deposited in the ISfinder database [74].

## 4. Conclusions

This study has provided an insight into the overall structure, genetic load, and possible ecological role of plasmid pP32BP2 from the psychrophilic *Psychrobacter* sp. DAB_AL32B. Plasmid pP32BP2 is the second-largest extrachromosomal replicon identified so far in the genus *Psychrobacter*. A significant part of the plasmid genome is comprised of three genetic modules (FIM, CAR and CAI), but only the first two were shown to be functional.

The FIM module encodes structural proteins and components required for the assembly of fimbriae via the chaperone-usher pathway. Using a crystal-violet staining assay, we demonstrated that this module positively influences the attachment of DAB_AL32B cells to abiotic surfaces. Moreover, we showed that the FIM module is functional in heterologous hosts representing various classes of *Proteobacteria*.

The CAR module has a unique gene arrangement among *Psychrobacter* spp. Homologous gene clusters are prevalent in closely related *Acinetobacter* spp., which suggests the origin of this plasmid-borne module and the direction of its horizontal transmission. The CAR modules (e.g., those found in *Acinetobacter* spp.) determine the aerobic breakdown of carnitine, whereby this compound is metabolized (with the release of the by-product TMA) and used as a carbon source. In such circumstances, the presence of pP32BP2 (carrying the CAR module) should be beneficial for the strain DAB_AL32B, since it could broaden the spectrum of metabolically available compounds. However, we showed that the TMA produced is toxic for DAB_AL32B cells, and it prevents their growth when carnitine is present in the medium. This phenomenon makes pP32BP2 a unique and interesting example of an extrachromosomal replicon which can bring benefits and drawbacks to its bacterial host depending on the environmental conditions and specific metabolic properties of the strain (i.e., ability to utilize toxic TMA).

## Figures and Tables

**Table 1:** Complete transposable elements encoded within plasmid pP32BP2.

Gene(s)	Transposable Element	Family/GROUP	Size (bp)	IR Sequence ^1,2,3^	DR Sequence ^1^
*pP32BP2_p04*	IS*Pssp4*	IS*256*/-	1302	IRL: GGGGGTTTCCTAAAAAC TGTGTAACTGC IRR: GAGACCATCCCGAATTC TGTGTAACTGC	CTTAAAAA
*pP32BP2_p19*-*20*	IS*Pssp5*	IS*5*/IS*427*	837	IRL: GGGTGTGTCATCAATTA IRR: GGGCGTGTCATCAATTA	TA

^1^ Sequences are shown in the 5′ to 3′ orientation. ^2^ IRL—left IR; IRR—right IR. ^3^ Residues identical in IRL and IRR are indicated in bold.

**Table 2:** Plasmids used and constructed in this study.

Plasmid Name	Characteristics ^1^	Reference
pABW1	Km^r^; 4.5 kb; *ori* pMB1; Mob^+^; *oriT* RK2; *lacZ*α; MCS	[58]
pABW1-REPPAR	pABW1 carrying REP and PAR modules of pP32BP2 (PCR- amplified with primers L232BREP and R232BREP)	This work
pBBR1MCS-2	Km^r^; 5.1 kb; *ori* pBBR1; Mob^+^; *oriT* RK2; *lacZα*; MCS	[59]
pBBR-Ps	pBBR1MCS-2 carrying REP module of pP32BP2 (PCR-amplified with primers L32REP and R32REP)	This work
pBBR-Ps-CAR	pBBR-Ps carrying CAR module of pP32BP2 (coordinates 31,262–44,421) inserted between SacI and SalI sites	This work
pBBR-FIM	pBBR1MCS-2 carrying FIM module of pP32BP2 (coordinates 24,246–31,265) inserted between BamHI and SalI sites	This work
pBBR-Ps-FIM	pBBR-Ps carrying FIM module of pP32BP2 (coordinates 24,246–31,265) inserted between SacI and SalI sites	This work
pBGS18	Km^r^; 3.7 kb; *ori* pMB1; *lacZ*α; MCS	[60]
pRK2013	Km^r^; helper plasmid carrying genes for conjugal transfer of RK2	[61]

^1^ Primer sequences are listed in Table S1.

## Supplementary Materials

The following are available online at https://www.mdpi.com/1422-0067/20/8/2015/s1, Figure S1: Box plots of OD_570_/OD_600_ ratios for the results of the crystal violet staining test performed for *Psychrobacter* spp. wild-type, pP32BP2-less, and plasmid-less carrying pBBR-Ps-FIM strains as well as heterologous wild-type and FIM module-carrying strains; Figure S2: Comparison of the CAR module of pP32BP2 and related modules found in *Psychrobacter* and *Acinetobacter* genomes; Figure S3: Growth of *Psychrobacter* sp. DAB_AL32B wild-type and plasmid-less strains under anaerobic conditions on sodium succinate and carnitine; Table S1: Genes located within plasmid pP32BP2; Table S2: Primers used in this study.
