# Supplementary material for: Benefits and Drawbacks of Harboring Plasmid pP32BP2, Identified in Arctic Psychrophilic Bacterium Psychrobacter sp. DAB_AL32B
Source: Int J Mol Sci. 2019 Apr 24;20(8):2015. doi: 10.3390/ijms20082015 (PMC6514802; doi:10.3390/ijms20082015)
Supplement: Supplementary file 1 [file ijms-20-02015-s001.pdf]

International Journal of
Molecular Sciences

MDPI

Article

Benefits and Drawbacks of Harboring Plasmid pP32BP2, Identified in Arctic Psychrophilic Bacterium *Psychrobacter* sp. DAB_AL32B

Anna Ciok, Adrian Cegielski, Dariusz Bartosik and Lukasz Dziewit *

Department of Bacterial Genetics, Institute of Microbiology, Faculty of Biology, University of Warsaw, Miecznikowa 1, 02-096 Warsaw, Poland; aciok@biol.uw.edu.pl (A.Ci.); cegielskiadrian@yahoo.com.au (A.Ce.); bartosik@biol.uw.edu.pl (D.B.)

* Correspondence: ldziewit@biol.uw.edu.pl; Tel.: +48-225-541-406

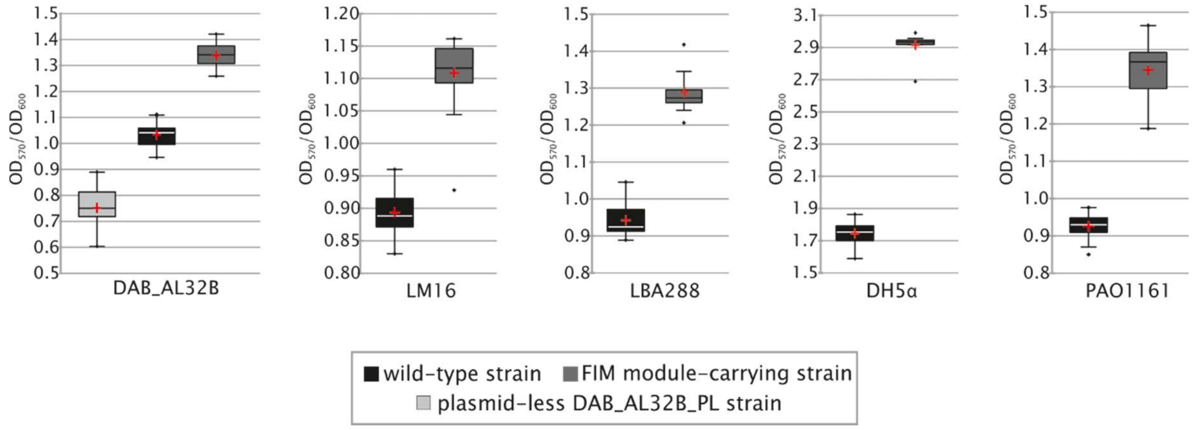

Figure S1. Box plots of OD_{570}/OD_{600} ratios for the results of the crystal violet staining test performed for *Psychrobacter* spp. wild-type, pP32BP2-less, and plasmid-less carrying pBBR-Ps-FIM strains as well as heterologous wild-type and FIM module-carrying strains. DAB_AL32B – *Psychrobacter* sp. DAB_AL32B, LM16 – *Achromobacter* sp. LM16, LBA288 – *A. tumefaciens* LBA288, DH5α – *E. coli* DH5α, PAO1161 – *P. aeruginosa* PAO1161. The red crosses correspond to the means. The central horizontal bars are the medians. The lower and upper limits of the box are the first and third quartiles, respectively. Points above and below the whiskers' upper and lower bounds are outliers.

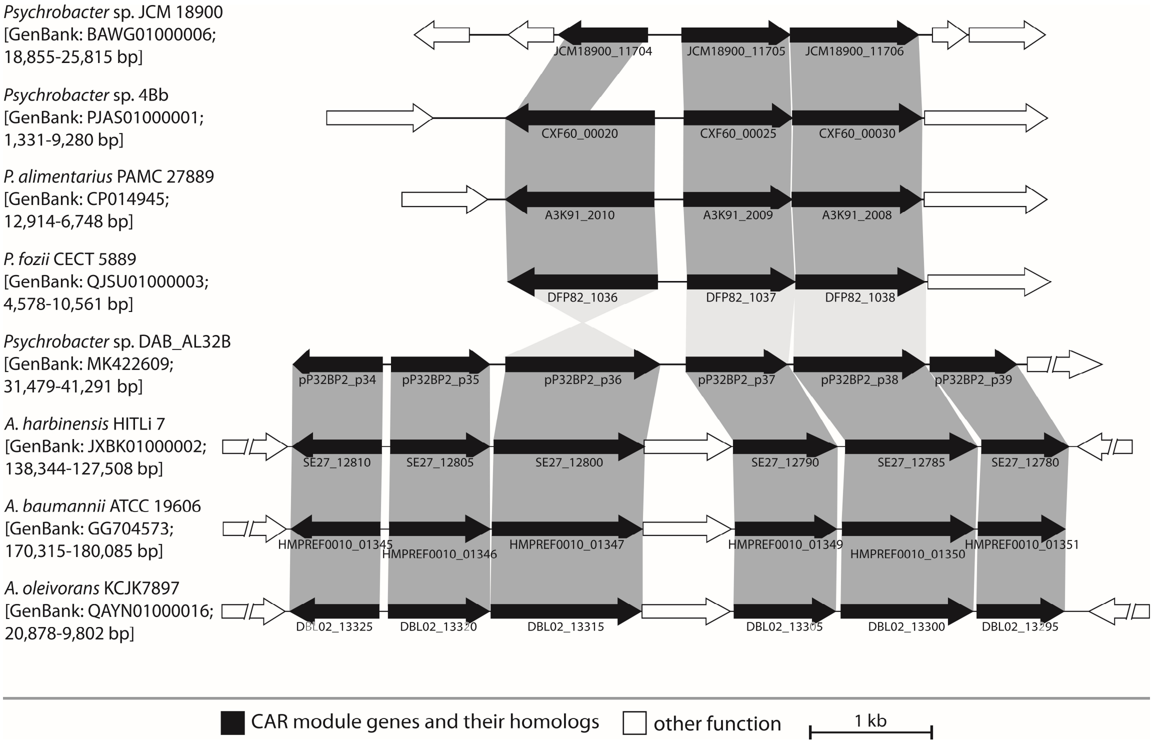

Figure S2. Comparison of the CAR module of pP32BP2 and related modules found in *Psychrobacter* and *Acinetobacter* genomes. Arrows indicate genes and their transcriptional orientation. GenBank accession numbers and coordinates of the gene clusters are shown in parentheses. Locus tags are shown below genes homologous to the CAR genes. The gray-shaded areas connect genes encoding proteins sharing ≥59% (dark gray) or 23–33% (light gray) aa identity, respectively.

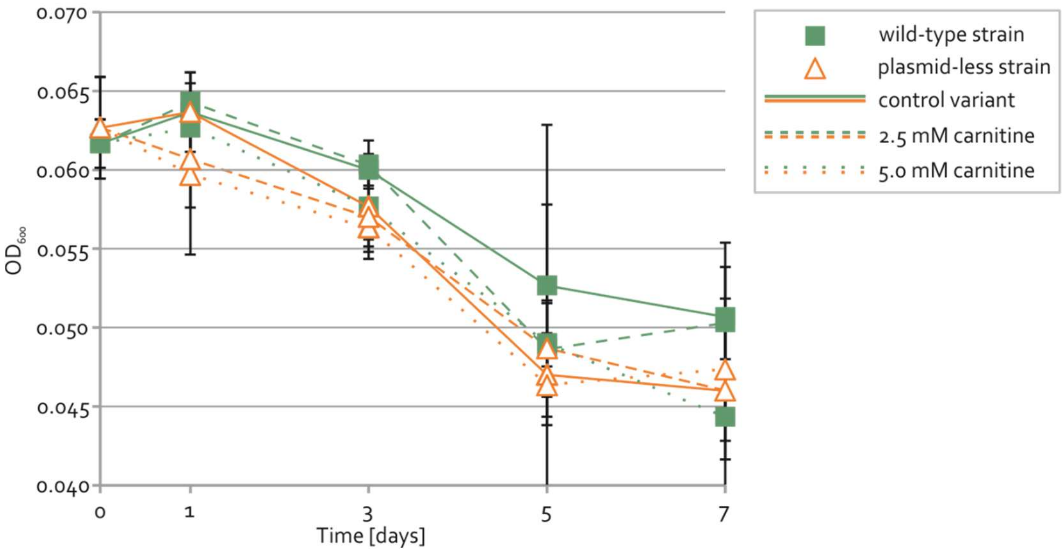

Figure S3. Growth of *Psychrobacter* sp. DAB_AL32B wild-type (green) and the plasmid-less (orange) strains under anaerobic conditions on sodium succinate (solid line) and carnitine (dashed and dotted lines, depending on final carnitine concentration). Control variant – bacteria cultivated in minimal medium supplemented with sodium succinate at a final concentration of 0.5% (w/v); 2.5 mM and 5.0 mM carnitine – bacteria cultivated in minimal medium additionally supplemented with carnitine at final concentrations of 2.5 or 5.0 mM, respectively. The mean values for three replicate cultures are plotted with error bars representing the standard deviations.

Table S1. Genes located within the pP32BP2 plasmid.

Gene	Coordinates	Protein length (aa)	Predicted function
pP32BP2_p01	571-1485	305	replication initiation protein RepA, COG5527
pP32BP2_p02	1654-2292	213	partitioning protein ParA, COG1192
pP32BP2_p03	2296-2505	70	partitioning protein ParB
pP32BP2_p04	2741-3955	405	transposase of IS*Pssp4*, IS*256* family, COG3328
pP32BP2_p05	4395-4057	112	hypothetical protein
pP32BP2_p06	4868-6043	392	putative competence protein, CoiA-like family, COG4469
pP32BP2_p07	6225-7148	308	hypothetical protein
pP32BP2_p08	7577-8167	197	serine recombinase, COG1961
pP32BP2_p09	8241-8486	82	transposase, IS*3* family, partial
pP32BP2_p10	9566-8553	337	glycosyltransferase, COG0463 [EC: 2.4.1.-]
pP32BP2_p11	10408-11988	527	Fic family protein, COG3177
pP32BP2_p12	12257-12523	89	transposase, IS*3* family, partial, COG2963
pP32BP2_p13	12475-13002	176	transposase, IS*3* family, partial
pP32BP2_p14	13027-13344	106	transposase, IS*3* family, partial, COG2801
pP32BP2_p15	18768-13498	1756	protein of unknown function
pP32BP2_p16	19844-19656	62	protein of unknown function (DUF1643)
pP32BP2_p17	20272-20039	77	transposase, IS*3* family, partial, COG2801
pP32BP2_p18	20448-20269	59	transposase, IS*3* family, partial
pP32BP2_p19	20559-20921	121	first part of transposase of IS*Pssp5*, IS*5* family, COG3293
pP32BP2_p20	20897-21307	145	second part of transposase of IS*Pssp5*, IS*5* family, COG3293
pP32BP2_p21	21557-21300	79	transposase, IS*3* family, partial, COG2801
pP32BP2_p22	21703-21542	53	transposase, IS*3* family, partial, COG2963
pP32BP2_p23	22050-21754	98	transposase, IS*3* family, partial, COG2963
pP32BP2_p24	22908-22174	244	hypothetical protein
pP32BP2_p25	23496-23669	58	transposase, IS*630* family, partial, COG3415
pP32BP2_p26	24110-23808	100	serine recombinase, partial, COG1961
pP32BP2_p27	24247-24074	57	serine recombinase, partial, COG1961
pP32BP2_p28	24403-24257	48	serine recombinase, partial, COG1961
pP32BP2_p29	24818-25348	177	major structural fimbrial subunit, COG3539
pP32BP2_p30	25427-26152	242	fimbriae assembly chaperone, COG3121
pP32BP2_p31	26223-28778	852	outer membrane usher protein, COG3188
pP32BP2_p32	28775-29791	339	putative adhesin
pP32BP2_p33	30346-30065	93	hypothetical protein
pP32BP2_p34	32462-31479	327	transcriptional regulator, LysR family, COG0583
pP32BP2_p35	32563-33651	363	malate dehydrogenase, COG0473 [EC:1.1.1.38]
pP32BP2_p36	33826-35532	569	carnitine transporter, BCCT (betaine/carnitine/choline) family, COG1292 [TC:2.A.15]
pP32BP2_p37	35813-36928	372	two component carnitine monooxygenase, oxygenase component, COG4638 [EC:1.14.13.239]
pP32BP2_p38	37003-38454	484	malic semialdehyde dehydrogenase, COG1012 [EC:1.2.1.4]
pP32BP2_p39	38499-39458	320	two component carnitine monooxygenase, reductase component, COG1018 [EC:1.14.13.239]
pP32BP2_p40	39579-41291	571	acetolactate synthase, large subunit, COG0028 [EC:2.2.1.6]
pP32BP2_p41	41518-42528	337	NADPH:quinone oxidoreductase, COG0604 [EC:1.6.5.5]
pP32BP2_p42	42655-44166	504	betaine aldehyde dehydrogenase, COG1012 [EC:1.2.1.8]
pP32BP2_p43	45257-44430	275	transcriptional regulator, IclR family, COG1414
pP32BP2_p44	45405-46589	395	crotonbetainyl-CoA reductase, COG1960 [EC:1.3.8.13]
pP32BP2_p45	46765-48003	413	γ-butyrobetainyl-CoA:carnitine CoA transferase, COG1804 [EC:2.8.3.16]
pP32BP2_p46	48119-48382	88	transposase, IS*3* family, partial, COG2963
pP32BP2_p47	48355-48603	83	transposase, IS*3* family, partial

pP32BP2_p48	48587-49066	160	transposase, IS*3* family, partial, COG2801
pP32BP2_p49	49115-49285	57	putative transposase, IS*3* family, partial
pP32BP2_p50	49973-50317	115	transporter EmrE, COG2076 [TC:2.A.7.1]
pP32BP2_p51	50412-50567	52	transcriptional regulator, AcrR family, partial, COG1309
pP32BP2_p52	52114-51581	177	transposase, IS*1* family, partial, COG3677 and COG1662
pP32BP2_p53	54149-52899	416	hypothetical protein
pP32BP2_p54	54229-54438	70	XRE-family like protein

Table S2. Primers used in this study.

Primer	Sequence[1]	Position[2]
L32REP	ataccggtGCGAACCACTGTGAGTATTG	54,185-54,204
R32REP	ataccggtTTAATTCTATCGCCCGCCTG	1,510-1,491
L232BREP	caaccggtTCTCCACCAATACTTATCACAG	54,069-54,090
R232BREP	gaaccggtTAGGGTCTTGATCAACTAGAC	2,647-2,627
F1	GGCATGGTTGCTCAAGGTAG	28,605-28,624
R1	CTATTGTACTGGTGGTATGCCG	30,182-30,161
F2	GCGATTCGATGCCAATGACT	30,649-30,668
R2	GAGCTTAATTCTTCGCCTGC	32,381-32,362
F3	ACCACTTGCTGAGGCATTAG	37,305-37,324
R3	GGTAAGCTGTTCAACTTCGG	15,903-15,884

[1] Sequences are shown in the 5′ to 3′ orientation. Sequence not complementary to the plasmid are shown in lowercase. Introduced restriction sites are underlined.

[2] Position of primers in pP32BP2 plasmid.
